# Recovery of Abnormal ABR in Neonates and Infants at Risk of Hearing Loss

**DOI:** 10.1155/2017/7912127

**Published:** 2017-04-04

**Authors:** Ioannis Psarommatis, Charalampos Voudouris, Ioannis Kapetanakis, Faselida Athanasiadi, Konstantinos Douros

**Affiliations:** ^1^Department of Otorhinolaryngology, “P. & A. Kyriakou” Children's Hospital of Athens, Thivon & Levadias Str., 11527 Athens, Greece; ^2^Neonatal Intensive Care Unit, 2nd Department of Pediatrics, Athens University Medical School, “P. & A. Kyriakou” Children's Hospital of Athens, Thivon & Levadias Str., 11527 Athens, Greece; ^3^3rd Department of Pediatrics, Athens University Medical School, “Attikon” University General Hospital, 1 Rimini St., 12464 Athens, Greece

## Abstract

The purpose of this retrospective study is to present the clinical experience of a single institution on the recovery of ABR thresholds in a large population of neonates and infants at risk of hearing loss. Potential prognostic factors associated with this phenomenon were also investigated. Out of 2248 high risk infants, 384 had abnormal ABR at initial hearing evaluation and 168 of them had absent ABR or a threshold ≥80 dBnHL. From this subgroup, a significant percentage showed complete or partial recovery on reexamination (32.7% and 9.3%, resp.), performed 4–6 months later. The presence of normal otoacoustic emissions was associated with the ABR restoration on reexamination. Moreover, the very young age at the initial hearing screening seems to be related to higher probabilities of false positive ABR. The potential recovery of hearing in HR infants raises concerns about the very early cochlear implantation in HR infants less than one year. Such a treatment modality should be decided cautiously and only after obtaining valid and stable objective and subjective hearing thresholds. This holds especially true for infants showing an auditory neuropathy profile, as they presented a much greater probability of ABR recovery.

## 1. Introduction

It is largely accepted among physicians that sensorineural hearing loss is caused by irreversible damage to the hearing organ, showing a permanent or progressively deteriorating character.

However, there are reports of recovered hearing thresholds in children. These reports first appeared in the early 1980s, when changes of the hearing status could be validated for the first time by using the Auditory Brainstem Responses (ABR), as a newly introduced technique of hearing evaluation. At that time, researchers reported on the changes of ABR thresholds in jaundiced infants. Those changes concerned not only the restoration of prolonged latencies after therapy but also the appearance of normal waveforms in cases with completely absent responses [1–6]. More recently, the widespread application of objective hearing tests for clinical and screening purposes has facilitated the diagnosis of more cases with hearing restoration during infancy [7–15]. The observed improvement of hearing thresholds has been mainly attributed to delayed maturation/myelination of the auditory pathway.

A number of neonates and infants at risk of hearing loss who showed restoration of their objective hearing thresholds have been gathered in our institution during the last two decades. Some of them (years 1992–2008) have already been reported [14].

Our purpose is to present a single institution's experience on the restoration of objective hearing thresholds in high risk (HR) infants. We have carried out a multivariate analysis to determine whether there are any indicators that can predict the recovery of auditory function. Illustrative cases are presented.

## 2. Patients and Methods

We reviewed retrospectively the audiological charts of all HR neonates (*n* = 2248) who were admitted to the neonatal intensive care unit (NICU) of our institution between 1992 and 2014. All children had one or more risk factors for hearing loss, as they have been defined and modified by the American Joint Committee on Infant Hearing during the study years [16–19]. Exclusion criteria were not applied.

Audiologic evaluation included history taking, otoscopy, and ABR testing under natural or chloral hydrate-induced sleep. OAEs were also performed in all neonates and infants with abnormal ABR responses. Immittance measurements were carried out when indicated.

For ABR recordings, a Biologic Traveller Express unit (years 1992–2003) and a Biologic System AEP Version 1.3.0 (years 2004–2014) were used (Bio-Logic Systems Corp., Mundelein, Chicago, IL, USA). Alternating clicks at a rate of 31.1/sec were presented monaurally through a shielded TDH-39 headphone. Responses were collected using four silver cup electrodes and then were amplified (×100,000) and filtered (100–3,000 Hz); 2048 samples were averaged and replicated. A replicable waveform at 40 dBnHL within the expected latencies was considered as “normal” or “pass” response, suggesting a normal hearing threshold. Babies who failed to produce a “pass” response uni- or bilaterally were scheduled for reevaluation 4–6 months later. In some cases, a third ABR session was necessary. Any improvement of ABR threshold by ≥10 dBnHL, compared to the initial ABR test, was considered as “recovery.”

Click-evoked OAEs were measured by a regularly calibrated ILO88 Otodynamic Analyzer (Otodynamics Ltd., UK, V3.92 and V6) in its default settings [20, 21]. An infant probe was used in all cases. If the middle ear was free from disease, a second OAEs test was performed for the majority of the reexamined infants.

### 2.1. Statistical Analysis

In univariate analysis we used chi-square and *t*-test for categorical and continuous variables, respectively. For multivariate analysis we used a logistic regression model and created a binary outcome variable describing the improvement of ABRs in follow-up studies; it was based on whether improvement in ABRs was at least 10 dBnHL. As predictors in the model we used OAE responses, sex, and risk factors that have been reported in the literature to cause hearing loss in the neonatal period, such as intake of ototoxic medications, birth weight, hyperbilirubinemia requiring exchange transfusions, and severe birth asphyxia.

Continuous variables are expressed as mean ± SD. Results are expressed as odds ratios (OR) and their corresponding 95% confidence intervals (CI).

Prognostic significance of OAEs has been based on the TEOAEs recordings from 69 out of 107 infants included in the statistical analysis. For the rest of 38 children, OAEs data were either missed or unreliable (reproducibility < 60%; stimulus stability < 70%).

In order to categorically exclude all ABR threshold elevations due to pure conductive losses (middle ear fluid or external canal debris), only infants demonstrating ABR thresholds ≥80 dBnHL were included in the analysis.

This research was conducted according to the rules of the institutional ethical committee.

## 3. Results

### 3.1. Epidemiology and Hearing Evaluation

**Figure 1:**
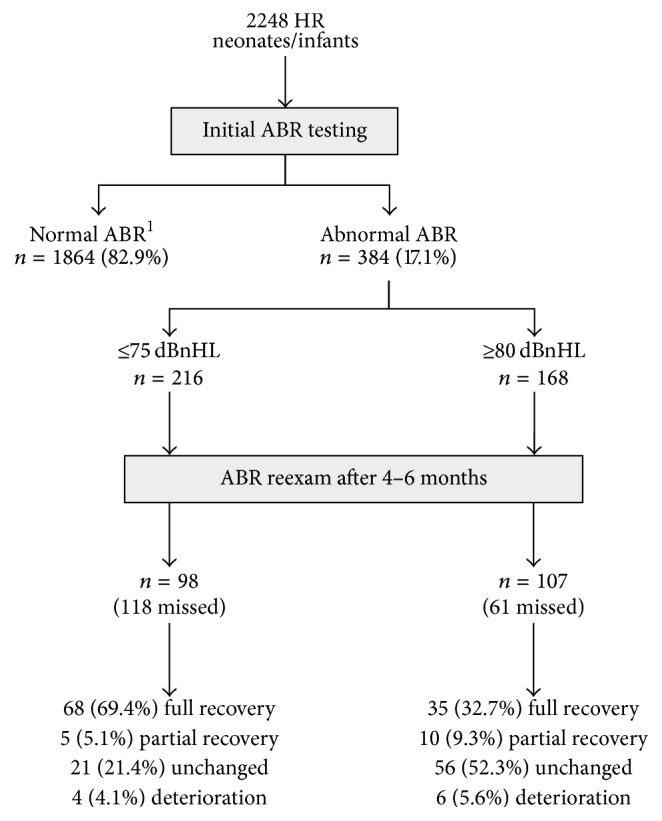
Schematic flow chart of the results of the study. ^1^Replicable waveform at 40 dBnHL within the expected latencies.

From the total HR population tested, 384 cases or 17.1% showed abnormal ABR findings, ranging from ABR elevation by 10 dBnHL to absent waveforms at maximum stimulation level (90/95 dBnHL), uni- or bilaterally, and they were scheduled for reexamination, after 4–6 months. Two hundred five appeared on follow-up assessment while 179 did not (53.4% versus 46.6%, resp.). From those presented, 103 showed complete ABR recovery, 15 showed partial recovery, and 87 remained stable or deteriorated (Figure 1). In these figures, all types and degrees of ABR elevation are included.

As most cases of mild to moderate hearing loss are expected to be due to middle ear problems, we focused on infants with initial ABR threshold ≥80 dBnHL through our statistical analysis. Overall, 168 infants showed ABR thresholds ≥80 dBnHL or no response at maximum stimulation level at initial screening. In this specific subgroup, 107 infants presented on reexamination and a substantial percentage of them showed full recovery to normal (32.7%) while 10 (9.3%) restored their ABR thresholds partially. From the same group, in 56 cases (52.3%) the ABR thresholds remained unchanged, whereas 6 (5.6%) cases showed threshold deterioration. The outcome of the hearing assessment is summarized in Figure 1.

**Figure 2:**
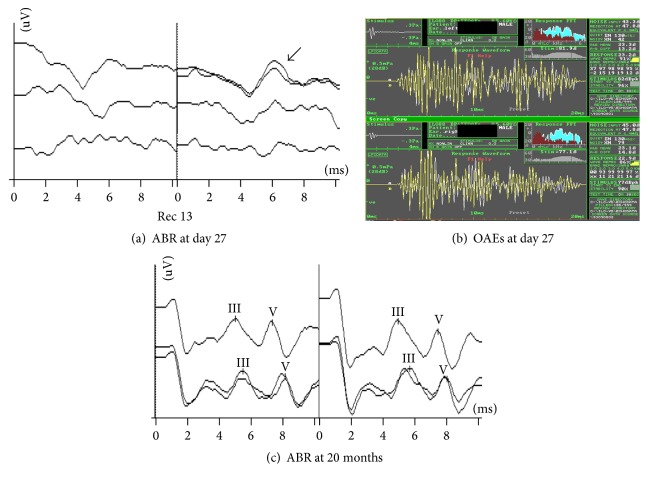
Audiological data from a HR infant of the early years of this study (risk indicators: prematurity, low birth weight, hyperbilirubinemia/phototherapy, and asphyxia). (a) Initial ABR recordings showing atypical waveforms at 90, 80, and 70 dBnHL (from above downwards). “Atypical” ABR waveforms were not a rare finding among infants suffering from AN who eventually showed full ABR restoration. We use the term “atypical” to describe any unexpected waveform, consisting of unpredictable yet reproducible waves. The black arrow denotes such a waveform. (b) Normal otoacoustic emissions were obtained bilaterally within the same session. (c) Last ABR testing at the age of 20 months. Typical and replicable waveforms were elicited at 60 and 40 dBnHL bilaterally (full ABR recovery). Information from the parents and behavioral audiometry validated the presence of normal hearing threshold.

**Figure 3:**
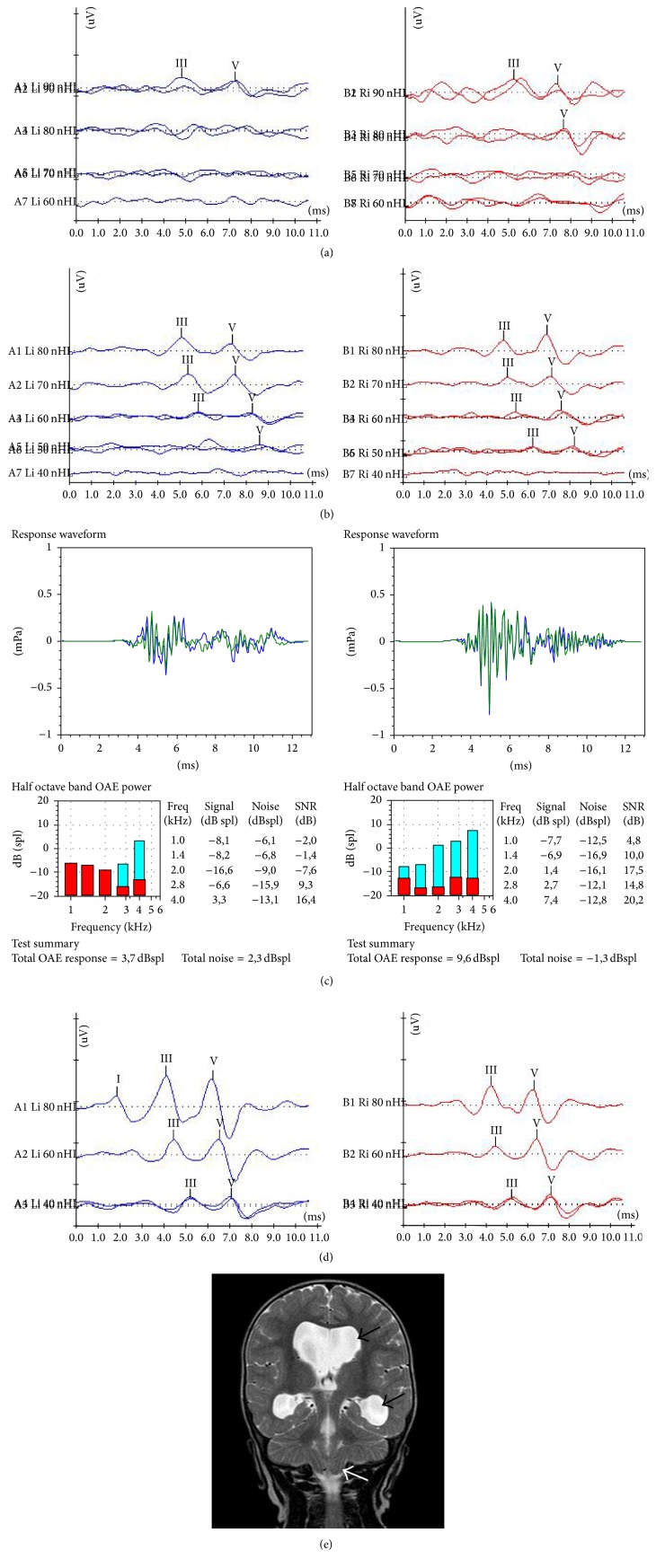
Serial ABR measurements, otoacoustic emissions and MRI findings of an infant with type I Chiari malformation. (a) ABR recordings at the initial hearing assessment (age of 2.5 months). Waveforms were obtained at 90 dBnHL on the left and 80 dBnHL on the right ear. (b) ABR findings 5 months later (age of 7.5 months). Waveforms were elicited bilaterally at 50 dBnHL. (c) At the same time, normal otoacoustic emissions were recorded on the right side and partial response on the left. (d) Last ABR session after 10 months (age of 18 months). Typical ABR waveforms were recorded at 40 dBnHL bilaterally, a finding which corresponds to “normal” ABR threshold and which is considered a strong indication of normal hearing. (e) Coronal MRI image of the same infant at age of 7 months, depicting enlargement of lateral ventricles (black arrows) and herniated cerebellar tonsils (white arrow). In this case, the ABR thresholds recovered completely.

Figures 2 and 3 are typical examples of severe auditory dysfunction recovery in the study population.

### 3.2. Statistical Analysis

One hundred seven infants with ABR thresholds ≥80 dBHL at the first measurement were finally included in our analysis. Their clinically important characteristics and the results of univariate analysis are shown in Table 1. The average age (corrected and chronological) at first hearing evaluation of HR infants who experienced full or partial recovery of their ABR thresholds was significantly lower than that of infants who did not show any recovery.

In multivariate analysis, only the presence of OAEs proved statistical significant predictor of ABR improvement (OR: 5.39, CI: 1.70–17.04, *p* = 0.004). Sex (OR: 1.35, CI: 0.41–4.45, *p* = 0.61), intake of ototoxic medications (OR: 0.86, CI: 0.25–2.95, *p* = 0.84), birth weight (OR: 1.00, CI: 0.99–1.01, *p* = 0.80), hyperbilirubinemia requiring exchange transfusions (OR: 4.15, CI: 0.64–26.89, *p* = 0.13), and existence of severe birth asphyxia (OR: 1.72, CI: 0.54–5.57, *p* = 0.36) were not statistically significant.

In our statistical analysis only infants with severely disturbed ABR thresholds (≥80 dBnHL) were included. However, there were infants with ABR thresholds between 45 and 75 dBnHL, who showed full or partial restoration of a purely sensorineural ABR abnormality. In these cases, a temporary conductive hearing loss could not be held responsible for this phenomenon because otoscopy and the presence of otoacoustic emissions confirmed that their middle ears were free of disease. These infants were excluded from the analysis on the basis of our inclusion criteria.

## 4. Discussion

Early hearing loss detection and intervention are paramount for the development of linguistic, cognitive, and social skills. Worldwide, thousands of hearing handicapped children enjoy the benefits of this approach, having attained educational and professional standards comparable to those of their normal hearing peers. Even today, however, and despite the substantial advances in diagnostic audiology, a valid diagnosis of hearing loss in neonates and infants presents considerable difficulties. The inherent weakness of objective tests to assess the real hearing thresholds, inability to employ subjective tests in many cases, middle ear problems, and comorbidities are the primary reasons for uncertainty in diagnosis. Moreover, low and mid frequency serviceable residual hearing can be hardly detected in the routine audiological evaluation of the very young child while pathologies with an auditory neuropathy profile further complicate the diagnostic process.

Apart from the above, a new confounding parameter has emerged from a number of reports; the potential of complete or partial recovery of auditory thresholds over time in neonates and infants with high risk factors for hearing loss. Such findings have been confirmed in several pediatric centers. Coenraad et al. [13] found significant improvement towards normal hearing or minimal hearing loss in 21.2% of HR infants with symmetric sensorineural hearing loss, including a child whose initial absent ABR recovered completely at follow-up examination. Hof et al. [15] reported similar findings in a number of preterm infants with initially abnormal ABR thresholds. Interestingly, 64% of their population showed full or partial recovery on reevaluation within the first year of life. They also reported cases with completely absent ABR at initial assessment, who eventually reached normal hearing levels. Six years ago, Psarommatis et al. [14] reported on the results of a targeted hearing screening program from a large series of HR infants. They detected reversible ABR abnormalities in 64% of children, regardless of the type and degree of ABR elevation. Impressively, 50% of the cases with absent waveforms or ABR threshold ≥80 dBnHL at initial screening recovered completely on reexamination within the first year. In the above-mentioned studies, researchers have mainly attributed the recovery of sensorineural loss to delayed maturation of the auditory system.

Except for the HR infants, complete or partial restoration of abnormal ABRs has also been reported in pediatric patients suffering from a variety of medical conditions, such as meningitis [22], metabolic diseases [23, 24], Cogan's syndrome [25], and auditory neuropathy [26, 27]. The present study adds a considerable number of infants showing the phenomenon of sensorineural hearing loss restoration to those already reported by other researchers. The observed ABR reversibility concerned not only mild/moderate threshold elevation but also severely increased or absent ABR as well.

To this point, we can only speculate the underlying etiology of this phenomenon. Delayed CNS maturation seems to be a reasonable explanation for HR neonates. Restoration of a normal CNS function after the resolution of harmful factors (e.g., asphyxia, jaundice, and CNS infection) could also justify such findings. Unfortunately, in these cases prognosis is impossible because no predictor for hearing recovery had been found so far.

Making use of our past experience on the possibility of ABR recovery in HR infants, in this study we included the presence of otoacoustic emissions to the potential predictors for partial or complete restoration of hearing in HR infants. In both uni- and multivariate analyses, the OAEs proved statistical significant predictor of ABR improvement, whereas the rest of potential predictors under investigation failed at this task. From a clinical viewpoint, it is much more likely that children who demonstrate typical otoacoustic emissions and abnormal or absent ABR at initial screening will show hearing improvement compared to those without OAEs.

Given that the combination of present OAEs in patients with abnormal ABR signifies an auditory neuropathy/dyssynchrony hearing pathology, we can state that, in HR infants showing an auditory neuropathy profile, partial or full ABR recovery may possibly take place in the following months. Specifically, we found that these infants have, on average, almost 5.5 times higher ABR recovery probabilities than those who do not show normal cochlear function at initial screening. Similar clinical reports but without statistical documentation have been described by other researchers, as well. Madden et al. observed improvement in behavioral thresholds over time in 9 out of 18 children with AN [27]. Psarommatis et al. concluded that in most HR infants fitting the profile of AN the ABR thresholds will be restored fully or partially during their infancy, using the term “transient infantile auditory neuropathy” to describe this phenomenon [28]. Attias and Raveh reported on 5 young candidates for cochlear implantation suffering from AN, all of which showed full or partial recovery on follow-up testing, 7–12 months later [26]. More recently, Harrison et al. found that one in five children with AN “…showed some threshold recovery to a level of hearing that allowed adequate speech understanding and language development without a hearing prosthesis” [29].

Therefore, there is sufficient evidence that the abnormal hearing thresholds can be improved or restored in some HR infants within a few months and this is especially true for infants suffering from hearing loss of auditory neuropathy type. The impact of such notion could be substantial for clinical practice.

The results in Figure 1 reveal that half of the “fail”/“refer” results of the initial screening recovered completely on reexamination (103 out of 205, for both ≥80 and ≤75 ABR elevation). This study also shows that the age (chronological or corrected) of the HR infants at the initial screening who displayed recovery of their severe ABR abnormalities is significantly lower as compared to the age of the infants with unchanged ABR (Table 1). This clinical observation—that the younger the age at the initial screening is, the higher the possibility for abnormal ABR findings appears to be—raises questions about the implementation time of the hearing screening in the high risk newborn. These findings led us to a modification of the applied hearing screening protocol over the last 4 years. Nowadays we administer the neonatal hearing screening for HR newborn/infants, not as soon as the health status of the newborn allows but at the age of 4–6 months. Refer cases are immediately subjected to complete diagnostic evaluation within the same session. This scheme has resulted in a dramatic decrease of false positives and reexaminations. Such a protocol allows sufficient time so that a possible middle ear or neural dysfunction has the chance of recovery while ensuring timely intervention, within the recommended timeframe. Although it may seemingly be against the current guidelines for early newborn hearing screening, by deferring the implementation time of the ABR screening 2-3 months later and definitely no later than the 6th month a much lower rate of false positives can be achieved. This, in turn, reduces the well-known drawbacks of early hearing screening, such as increased cost, unnecessary anxiety for parents, additional unneeded diagnostic tests, unfavorable labelling and discomfort for the child, and higher “loss to follow-up” rates. Obviously, any novel protocol should allow for the cases requiring immediate conservative or surgical treatment, such as CMV-infected infants or infants suffering from meningitis.

Moreover, the potential recovery of hearing in HR infants raises concerns about the very early cochlear implantation in children less than one year. Reports on very early cochlear implantation (5–12 months) have recently appeared in the medical literature [30–33]. Even if the anesthetic and surgical risk might be regarded as negligible, the diagnostic risk increases inversely proportional with age: the younger the child is the more likely a diagnostic error gets. To date, there are no means by which one can be sure about the real hearing threshold of a child less than 6 months. Thus, the audiological evaluation of a child cannot be considered “complete” at this age, so that uni- or bilateral cochlear implantation can be applied.

Although we favor the fastest auditory stimulation possible in the hard of hearing child, the intervention in the form of cochlear implantation in the HR infant should be recommended only after the ABR thresholds have been stabilized and reliable behavioral tests have been obtained. Infants suffering from diseases which can harm CNS (birth head trauma, hyperbilirubinemia, CNS infection, neurological and metabolic diseases, anoxia, etc.), sequential ABR, and imaging studies to monitor the progression of brain myelination are recommended. In such cases, the decision for cochlear implantation should not be based on an “early” ABR test. Instead, repeating the ABR test shortly before the cochlear implantation is strongly suggested.

Particularly, the HR infants with an auditory neuropathy profile should be treated less aggressively than the other HR infants, in view of the fact that partial or complete ABR recovery is more likely to take place in this group during the following months. In such cases, the indication for cochlear implantation should be made well after the first year of life and only after the subjective and objective hearing thresholds have been repeatedly confirmed. Bilateral cochlear implantation should probably be avoided within the first year in infants suffering from auditory neuropathy.

## 5. Conclusions

- A significant number of HR infants with absent ABR or ABR thresholds ≥80 dBnHL showed complete or partial recovery on reexamination (32.7% and 9.3%, resp.), after 4–6 months.
- The younger the age at first ABR evaluation is, the greater the likelihood of false positive results appears to be.
- Normal otoacoustic emissions recordings should be considered as an important predictor of ABR recovery since they remained significantly correlated with ABR recovery even after having taken into account a number of confounding factors.
- The decision for a very early cochlear implantation (<1 year) in HR infants should be made cautiously and only after obtaining valid and stable objective and subjective hearing thresholds.
- This is especially true for infants showing an auditory neuropathy profile, because they showed a much greater probability of partial or complete hearing recovery.
- Fast-track sequential or simultaneous bilateral cochlear implantation in HR infants younger than 1 year of age should be recommended only in exceptional cases with absolute indication.
- Any modification in the existing protocols should account for certain exceptions, such as neonates and infants with meningitis or CMV infection, which, according to the current level of knowledge, should be treated early.

## Figures and Tables

**Table 1:** Characteristics of patients in relation to the improvement of ABRs in follow-up measurements.

	ABR		*p* value
	Improvement	No improvement	
Sex (m/f)	27/18	35/27	0.84
Gestational age (weeks)	32.4 ± 4.9	34.1 ± 4.1	0.14
Corrected age^*∗*^ at first ABR (weeks)	3.4 ± 9.7	9.5 ± 8.3	0.001
Chronological age at first ABR (days)	109 ± 106	228 ± 271	0.010
Ototoxic medication (yes/no)	17/28	26/36	0.66
Birth weight (gr)	1975 ± 875	1980 ± 622	0.97
Exchange transfusions (yes/no)	7/38	12/50	0.79
Severe birth asphyxia (yes/no)	18/27	20/42	0.41
OAE (yes/no)	20/12	9/28	0.001

^*∗*^Corrected age = chronological age reduced by the number of weeks born before 40 weeks of gestation.
